# Effects of recombinant human brain natriuretic peptide in patients with acute myocardial infarction undergoing percutaneous coronary intervention: A systematic review and meta-analysis

**DOI:** 10.1097/MD.0000000000019479

**Published:** 2020-03-13

**Authors:** Cheng Ning, Yawei Zheng, Jie Li, Ming Liu, Zhuyuan Fang

**Affiliations:** a Affiliated Hospital of Nanjing University of Chinese Medicine; b Nanjing University of Chinese Medicine, Nanjing, Jiangsu, China.

**Keywords:** acute myocardial infarction, cardiac function, Meta-analysis, percutaneous coronary intervention, recombinant human brain natriuretic peptide

## Abstract

**Objective:**

To systematically review the effect of recombinant human brain natriuretic peptide (rhBNP) on the cardiac function in patients with acute myocardial infarction (AMI) undergoing percutaneous coronary intervention (PCI).

**Methods:**

PubMed, Web of Science, the Cochrane Library, Chinese Biomedical Database (CBD), and China National Knowledge Infrastructure (CNKI) were electronically searched to collect randomized controlled trials (RCTs) of traditional exercise for patients with AMI undergoing PCI from the beginning of the database inception to January 2019. Two reviewers independently screened the literature, extracted data, and evaluated the quality of included studies. Then, meta-analysis was performed using the RevMan 5.3 software.

**Results:**

A total of 16 RCTs and 1551 patients were included. The results of the meta-analysis showed that, compared with the control-treated patients, rhBNP-treated patients with AMI had an increased left ventricular ejection fraction (LVEF) of 3.34% ([MD = 3.34, 95% CI (0.39,6.29), *P* = .03]) 1 week postoperatively, 6.22% ([MD = 6.22, 95% CI (4.15,8.28), *P* < .00001]) 4 weeks postoperatively, 7.34% ([mean difference (MD) = 7.34, 95% CI (4.52, 10.16), *P* < .00001]) 12 weeks postoperatively, and 5.32% ([MD = 5.32, 95% CI (3.05, 7.59), *P* < .00001]) 24 weeks postoperatively. Moreover, the heart failure (HF) recurrence of rhBNP-treated patients with AMI 12 weeks postoperatively was 0.24 times that of the control-treated patients ([risk ratio (RR) = 0.24, 95% CI (0.06, 0.92), *P* = .04]), and the difference was statistically significant. At the same time, rhBNP-treated patients had decreased N-terminal pro-brain natriuretic peptide (NT-proBNP) (24 hours, 48 hours, 72 hours) and aldosterone (Ald) (24 hours, 72 hours, 168 hours) levels in comparison with the control-treated patients.

**Conclusion:**

Current evidence shows that the application of rhBNP presents a greater clinical benefit to patients with AMI undergoing PCI. Due to the methodological bias in the included studies and small sample size, more high-quality studies are required to verify the study findings.

**Systematic Review Registration Number:**

PROSPERO (CRD42019126727)

## 1 Introduction

The incidence and mortality of acute myocardial infarction (AMI) worldwide has increased significantly in recent years. Acute heart failure (HF) is a serious complication of AMI, being one of the most important causes of death in myocardial infarction. Ventricular failure after AMI remains the most common cause of cardiogenic shock, accounting for more than 80% of cases. However, with the emergence of multiple treatments such as percutaneous coronary intervention (PCI), the mortality rate of AMI has dropped from 20% in the late 1980s to 5% to 7% at present.^[1–4]^ Despite significant progress in the management of myocardial infarction by direct PCI, its morbidity and mortality remain significant. Improving patient outcomes and reducing AMI mortality are still the focus of clinical management. With the progress of interventional therapy, the mortality rate of patients with AMI complicated with acute HF has decreased; however, acute HF is still the primary cause and the most serious complication resulting in hospital death in AMI. Brain natriuretic peptide is an endogenous hormone that is mainly synthesized and secreted by left ventricular cardiomyocytes. It has the effects of sodium and diuretics, reducing vascular tone and antagonizing the renin-angiotensin-aldosterone (Ald) system and sympathetic nervous system activity.^[5]^ Lyophilized recombinant human brain natriuretic peptide (rhBNP) can mimic the action of endogenous brain natriuretic peptide, and its clinical application in HF has become the central focus of research.^[6]^

There is increasing evidence that rhBNP can significantly improve the prognosis of patients with AMI.^[7]^ We initiated a systematic review and meta-analysis that aimed to collect, validate, and reanalyse the effects of rhBNP in patients with AMI undergoing PCI. This study was registered at PROSPERO (CRD42019126727) and conducted in compliance with the PRISMA (Preferred Reporting Items for Systematic Reviews and Meta-analyses) guidelines.^[8]^

## 2 Materials and methods

### 2.1 Ethical considerations

All analyses were based on previous published studies, thus no ethical approval and patient consent are required.

### 2.2 Literature retrieval

The following electronic databases were carefully searched focusing on rhBNP therapy in patients with AMI undergoing PCI: PubMed, Web of Science, the Cochrane Library, Chinese Biomedical Database (CBD), and China National Knowledge Infrastructure (CNKI). The search terms employed were as follows: (‘recombinant human brain natriuretic peptide’ OR ‘recombinant human B-type natriuretic peptide’ OR ‘rhBNP’ OR ‘nesiritide’ OR ‘Natrecor’) AND (‘Myocardial Infarction’ OR ‘acute myocardial infarction’ OR ‘AMI’) AND (‘percutaneous coronary intervention’ OR ‘PCI’ OR ‘coronary artery stent implantation’), along with a combination of MeSH indexing approaches. The search included publications from the beginning of the database inception to January 2019, without restrictions by language or publication status. Published studies listed in the references of eligible reports were also screened to avoid possible omissions.

### 2.3 Selection criteria

The eligible publications were determined as to whether they satisfied the following inclusion criteria:

(1) articles published in any language using human samples;
(2) randomized controlled trials (RCTs);
(3) data related to the application of rhBNP in patients with AMI undergoing PCI;
(4) in the control group, patients were treated with conventional therapy, such as anti-freeing, antiplatelet, nitrates, angiotensin-converting enzyme inhibitors, angiotensin-receptor blockers, etc.;
(5) in the rhBNP group, rhBNP was injected intravenously after PCI on the basis of control group treatment;
(6) studies must provide original and complete data;
(7) study with the latest or most complete data was selected when >1 article using the same samples was published.

The exclusion criteria were as follows:

(1) repeated publication of literature,
(2) duplicated data, and
(3) reports with incomplete data or no usable data.

### 2.4 Data extraction

For eligible studies, the following data were extracted based on the inclusion criteria: name of the first author, year of publication, sample sizes, levels of left ventricular ejection fraction (LVEF), N-terminal pro-brain natriuretic peptide (NT-proBNP), Ald, HF recurrence, and follow-up observations. Disagreements were discussed or presented to a third reviewer until a consensus was reached. Data from each article were independently collected by 2 investigators.

### 2.5 Quality assessment

The reviews independently assessed the quality of the included studies using the Cochrane Handbook for Systematic Reviews of Intervention.^[9]^ Articles were evaluated based on 7 parameters: ‘random sequence generation,’ ‘allocation concealment,’ ‘blinding of participants and personnel,’ ‘blinding of outcome assessment,’ ‘incomplete outcome data,’ and ‘selective reporting; other bias.’ The terms ‘low risk of bias,’ ‘unclear risk of bias,’ and ‘high risk of bias’ were used to characterize the quality.

### 2.6 Outcome variables

The primary efficacy outcome of the present study was the LVEF value. The secondary endpoint outcome was HF recurrence. Additional outcomes of interest included NT-proBNP levels and Ald levels. Endpoint outcomes were evaluated at different follow-up observations, according to data from the included studies.

### 2.7 Statistical analysis

The Review Manager version 5.3 software (The Cochrane Collaboration, Copenhagen, Denmark) was used to perform the statistical analysis. Heterogeneity among the same category was evaluated using the χ2 test and Cochran's Q statistic: if *P* > .1 and I^2^ ≤ 50%, the probability of heterogeneity was considered to be low, and the fixed-effects model would be used; otherwise, the random-effect model was used.^[10]^ Continuous data were analysed based on the weighted mean difference (MD) if the variables measured were the same; otherwise, they were analysed based on the standardized mean difference (SMD). Count data were analysed using the risk ratio (RR). The 95% confidence intervals (CIs) were calculated for all the analyses. The inverse-variance model was used to calculate pooled MDs and SMDs for continuous data, while the Mantel-Haenszel model was used to calculate pooled RRs for count data. The pooled MDs, SMDs, and RRs were measured using the *Z* test, and a *P* value of <.05 was considered statistically significant. We performed a visual estimation of a funnel plot to evaluate the possibility of publication if more than 5 studies were included for the outcome.^[11]^ We carried out sensitivity analysis to detect the individual effect of each study by omitting one individual interstudy at a time.

## 3 Results

### 3.1 Study characteristics

**Figure 1:**
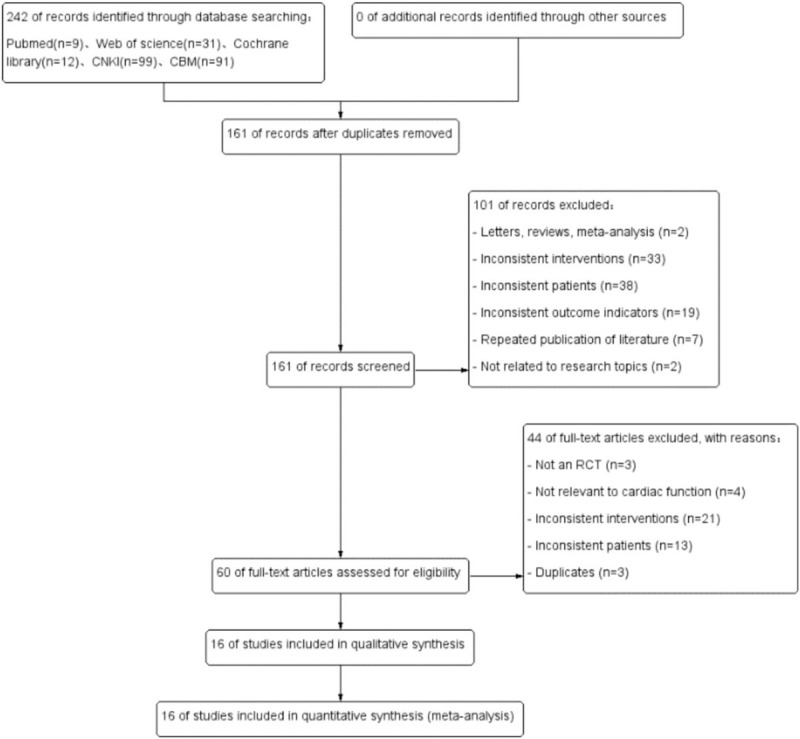
Flow diagram of the literature selection.

**Figure 2:**
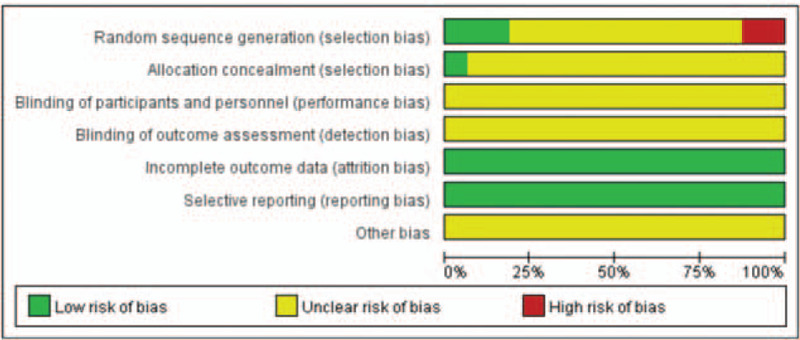
Risk of bias summary and graph.

A total of 242 potentially relevant articles were retrieved from the initial search, according to the previously mentioned inclusion criteria (Fig. 1). Ultimately, analyses of 16 RCTs^[12–27]^ involving 1551 patients with AMI after PCI were performed in this meta-analysis, including 775 patients in the rhBNP group and 776 patients in the control group. The major characteristics of the included studies are shown in Table 1. One of 16 studies had multicenter designs.^[23]^ Standard medical therapy was prescribed to all patients irrespective of the assigned treatment. The risk for bias of the included studies is shown in Figure 2.

**Table 1:**
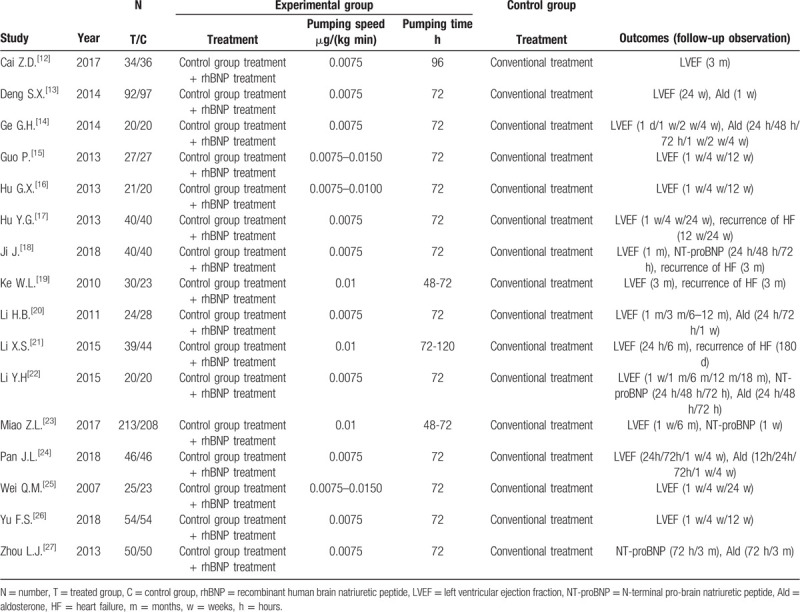
Characteristics of the included studies.

### 3.2 Meta-analysis results

#### 3.2.1 Analysis of LVEF

**Figure 3:**
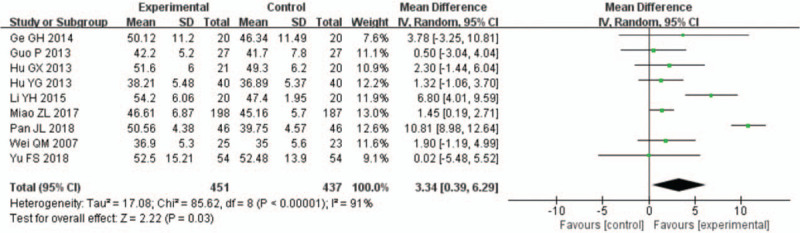
Forest plots for the comparison of left ventricular ejection fraction (1 week after percutaneous coronary intervention).

**Figure 4:**
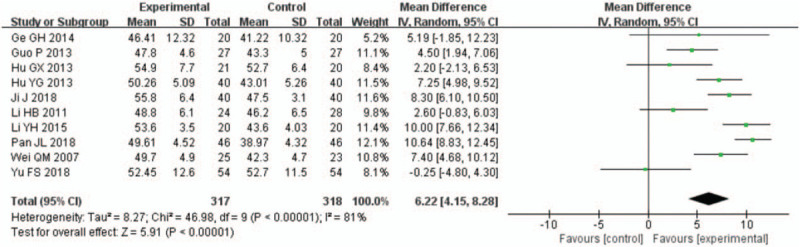
Forest plots for the comparison of left ventricular ejection fraction (4 weeks after percutaneous coronary intervention).

**Figure 5:**
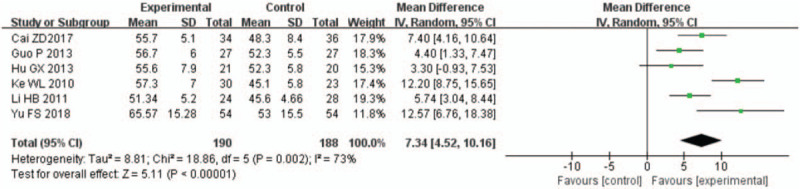
Forest plots for the comparison of left ventricular ejection fraction (12 weeks after percutaneous coronary intervention).

**Figure 6:**
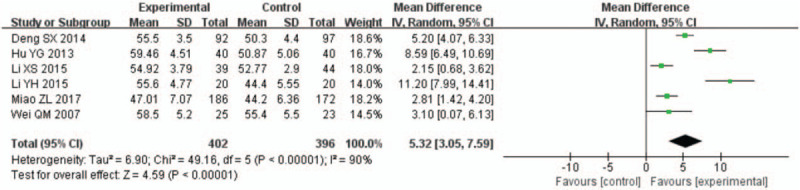
Forest plots for the comparison of left ventricular ejection fraction (24 weeks after percutaneous coronary intervention).

A total of 15 studies^[12–26]^ reported on the LVEF of patients with AMI undergoing PCI. The follow-up observations were conducted 1 week, 4 weeks (or 1 month), 12 weeks (or 3 months), and 24 weeks (or 6 months) after PCI. All data displayed high heterogeneity, and the meta-analysis results showed that the LVEF was significantly higher in the rhBNP group than in the control group (Figs. 3–6). In 9 studies with 451 rhBNP patients and 437 control patients, the LVEF of patients in the rhBNP group was significantly higher ([MD = 3.34, 95% CI (0.39,6.29), *P* = .03]) than that in the control group 1 week after PCI (Fig. 3). In 10 studies with 317 rhBNP patients and 318 control patients, the LVEF of patients in the rhBNP group was significantly higher ([MD = 6.22, 95% CI (4.15, 8.28), *P* < .00001]) than that in the control group 4 weeks after PCI (Fig. 4). In 6 studies with 190 rhBNP patients and 188 control patients, the LVEF of patients in the rhBNP group was significantly higher ([MD = 7.34, 95% CI (4.52, 10.16), *P* < .00001]) than that in the control group 12 weeks after PCI (Fig. 5). In 6 studies with 402 rhBNP patients and 396 control patients, the LVEF of patients in the rhBNP group was significantly higher ([MD = 5.32, 95% CI (3.05, 7.59), *P* < .00001]) than that in the control group 24 weeks after PCI (Fig. 6).

#### 3.2.2 Analysis of HF recurrence

**Figure 7:**
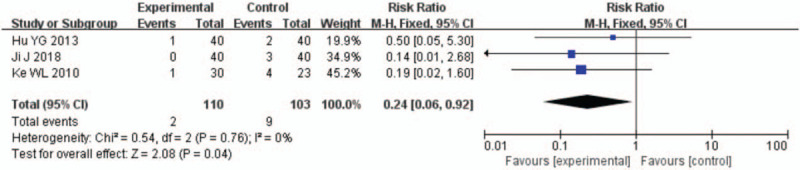
Forest plots for the comparison of heart failure recurrence (12 weeks after percutaneous coronary intervention).

A total of 3 studies^[17–19]^ reported on the HF recurrence in patients with AMI undergoing PCI. The follow-up observation was 12 weeks (or 3 months) after PCI. All data displayed high homogeneity, including 3 studies with 110 rhBNP patients and 103 control patients, and the meta-analysis results showed that the difference ([RR = 0.24, 95% CI (0.06, 0.92), *P* = .04]) between the rhBNP group and the control group was statistically significant 3 months postoperatively (Fig. 7).

#### 3.2.3 Analysis of NT-proBNP

**Figure 8:**
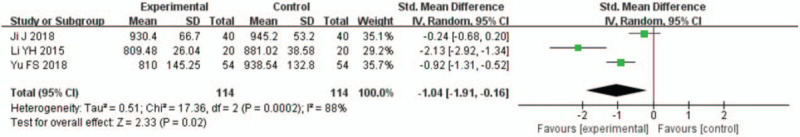
Forest plots for the comparison of N-terminal pro-brain natriuretic peptide (24 hours after percutaneous coronary intervention).

**Figure 9:**
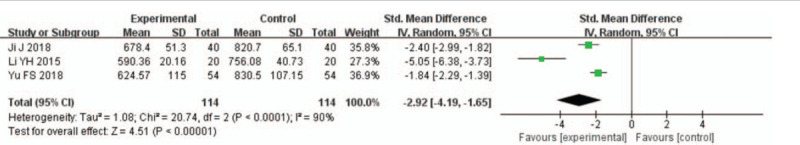
Forest plots for the comparison of N-terminal pro-brain natriuretic peptide (48 hours after percutaneous coronary intervention).

**Figure 10:**
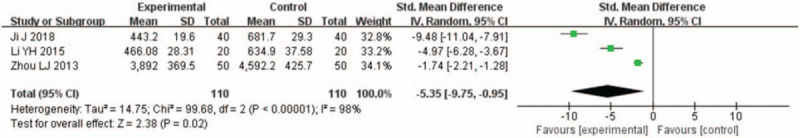
Forest plots for the comparison of N-terminal pro-brain natriuretic peptide (72 hours after percutaneous coronary intervention).

A total of 4 studies^[18,22,23,27]^ reported on the NT-proBNP levels of patients with AMI undergoing PCI. The follow-up observations were conducted 24 hours, 48 hours, and 72 hours after PCI. All data displayed high heterogeneity, and the meta-analysis results showed that the NT-proBNP levels were significantly lower in the rhBNP group than in the control group (Figs. 8–10). In 3 studies with 114 rhBNP patients and 114 control patients, the NT-proBNP levels of patients in the rhBNP group were significantly lower ([SMD = −1.04, 95% CI (−1.91,−0.16), *P* = .04]) than those in the control group 24 hours after PCI (Fig. 8). In 3 studies with 114 rhBNP patients and 114 control patients, the NT-proBNP levels of patients in the rhBNP group were significantly lower ([SMD = −2.92, 95% CI (−4.19,−1.65), *P* < .00001]) than those in the control group 48 hours after PCI (Fig. 9). In 3 studies with 110 rhBNP patients and 110 control patients, the NT-proBNP levels of patients in the rhBNP group were significantly lower ([SMD = −5.35, 95% CI (−9.75,−0.95), *P* = .04]) than those in the control group 72 hours after PCI (Fig. 10).

#### 3.2.4 Analysis of Ald levels

**Figure 11:**
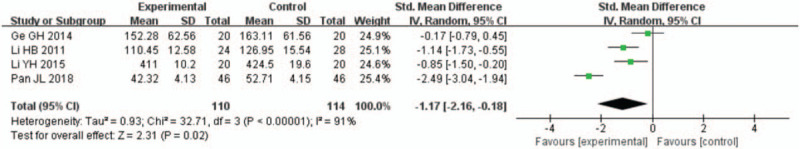
Forest plots for the comparison of aldosterone (24 hours after percutaneous coronary intervention).

**Figure 12:**
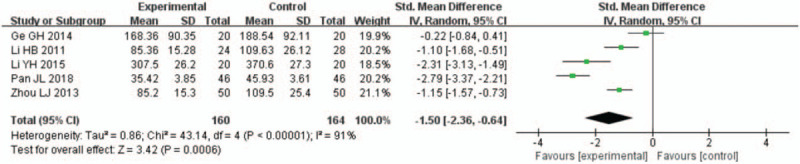
Forest plots for the comparison of aldosterone (72 hours after percutaneous coronary intervention).

**Figure 13:**
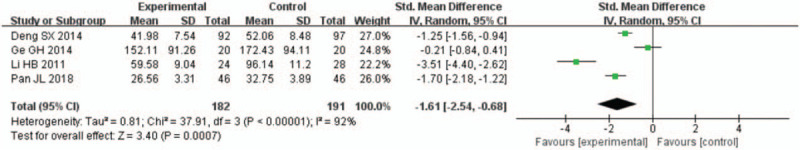
Forest plots for the comparison of aldosterone (168 hours after percutaneous coronary intervention).

A total of 6 studies^[13,14,20,22,24,27]^ reported on the Ald levels of patients with AMI undergoing PCI. The follow-up observations were conducted 24 hours, 72 hours, and 168 hours after PCI. All data displayed high heterogeneity, and the meta-analysis results showed that the Ald levels were significantly lower in the rhBNP group than in the control group (Figs. 11–13). In 4 studies with 110 rhBNP patients and 114 control patients, the Ald levels of patients in the rhBNP group were significantly lower ([SMD = −1.17, 95% CI (−2.16, −0.18), *P* = .02]) than those in the control group 24 hours after PCI (Fig. 11). In 5 studies with 160 rhBNP patients and 164 control patients, the Ald levels of patients in the rhBNP group were significantly lower ([SMD = −1.50, 95% CI (−2.36, −0.64), *P* = .0006]) than those in the control group 72 hours after PCI (Fig. 12). In 4 studies with 182 rhBNP patients and 191 control patients, the Ald levels of patients in the rhBNP group were significantly lower ([SMD = −1.61, 95% CI (−2.54, −0.68), *P* = .0007]) than those in the control group 168 hours after PCI (Fig. 13).

#### 3.2.5 Sensitivity analysis and publication bias

No single study significantly altered the outcomes of our meta-analysis when each individual study was omitted, suggesting that the results were stable and robust.

**Figure 14:**
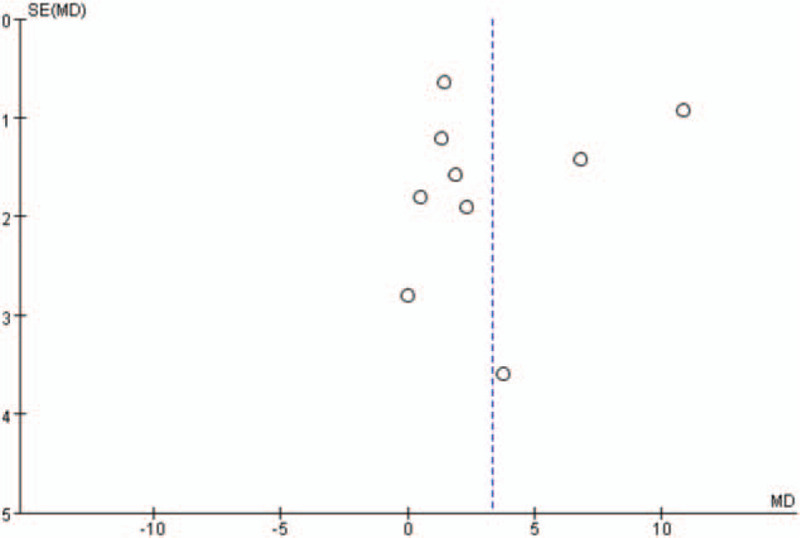
Funnel plots for the comparison of left ventricular ejection fraction (1 week after percutaneous coronary intervention).

**Figure 15:**
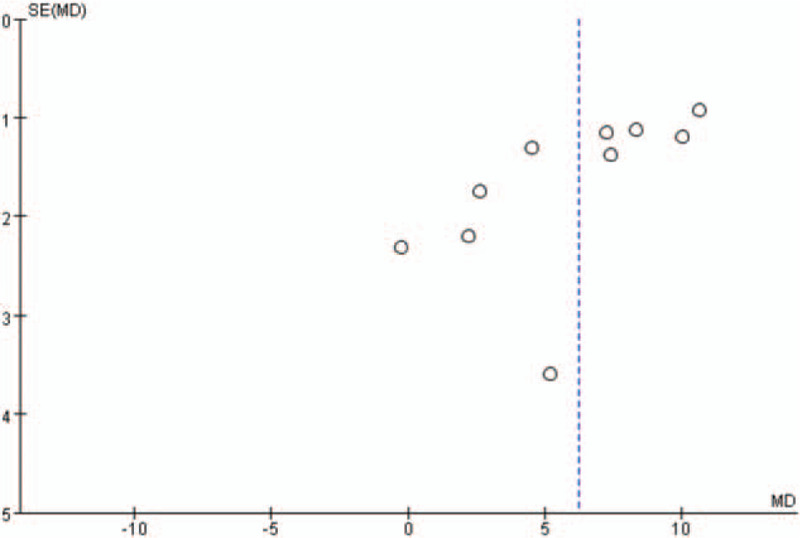
Funnel plots for the comparison of left ventricular ejection fraction (4 weeks after percutaneous coronary intervention).

**Figure 16:**
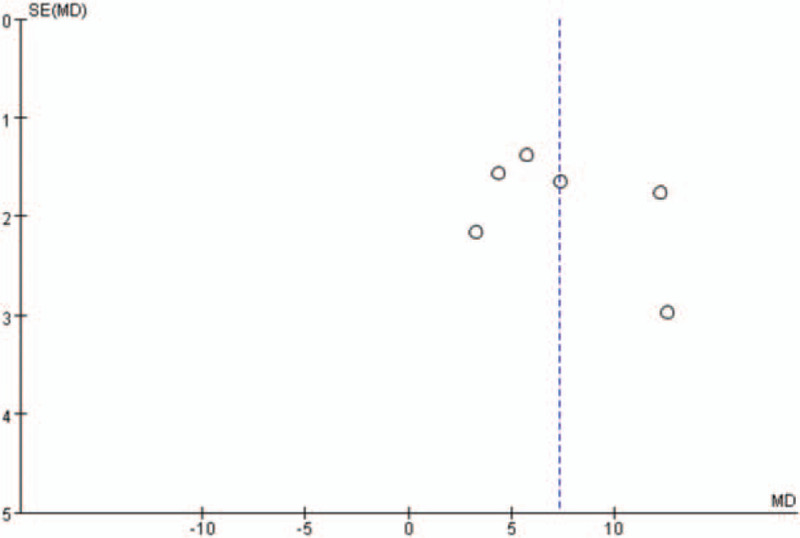
Funnel plots for the comparison of left ventricular ejection fraction (12 weeks after percutaneous coronary intervention).

**Figure 17:**
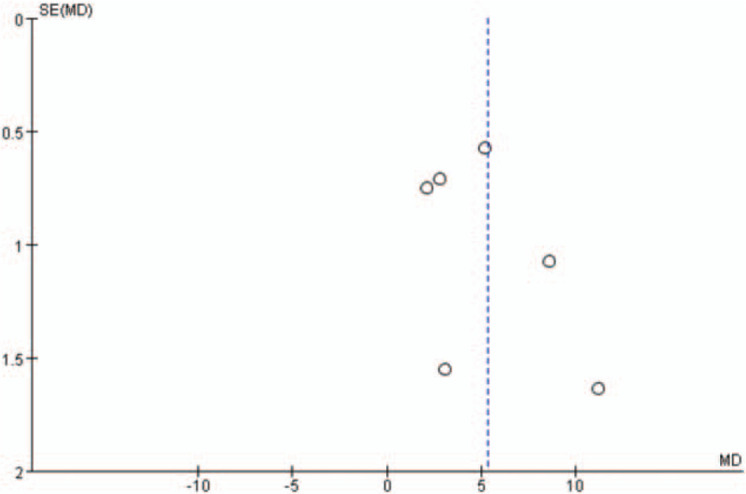
Funnel plots for the comparison of left ventricular ejection fraction (24 weeks after percutaneous coronary intervention).

Additionally, the shape of the funnel plots in the LVEF analysis, with more than 5 studies included, did not show any obviously asymmetrical evidence as presented in Figures 14–17. As a result, we suggested that there was a little evidence of publication bias observed in the LVEF analysis.

## 4 Discussion

The present meta-analysis compared the clinical outcomes of rhBNP and control treatment in the management of patients with AMI undergoing PCI. Our major finding revealed that, compared with the control-treated patients, rhBNP-treated patients with AMI had an increased LVEF of 3.34% ([MD = 3.34, 95% CI (0.39,6.29), *P* = .03]) 1 week postoperatively, 6.22% ([MD = 6.22, 95% CI (4.15,8.28), *P* < .00001]) 4 weeks postoperatively, 7.34% ([MD = 7.34, 95% CI (4.52, 10.16), *P* < .00001]) 12 weeks postoperatively, and 5.32% ([MD = 5.32, 95% CI (3.05, 7.59), *P* < .00001]) 24 weeks postoperatively. Furthermore, the HF recurrence of rhBNP-treated patients with AMI 12 weeks postoperatively was 0.24 times that of the control-treated patients ([RR = 0.24, 95% CI (0.06, 0.92), *P* = .04]), and the difference was statistically significant. At the same time, rhBNP-treated patients had decreased NT-proBNP levels (24 hours: [SMD = −1.04, 95% CI (−1.91,−0.16), *P* = .04]; 48 h: [SMD = −2.92, 95% CI (−4.19,−1.65), *P* < .00001]; 72 hours: [SMD = −5.35, 95% CI (−9.75,−0.95), *P* = .04]) and Ald levels (24 hours: [SMD = −1.17, 95% CI (−2.16, −0.18), *P* = .02]; 72 hours: [SMD = −1.50, 95% CI (−2.36, −0.64), *P* = .0006]; 168 hours: [SMD = −1.61, 95% CI (−2.54, −0.68), *P* = .0007]) in comparison with the control-treated patients.

This study evaluated the effects of rhBNP on cardiac function in patients with AMI treated with emergency PCI. AMI leads to a widespread ischemic necrosis of cardiomyocytes, which reduces myocardial contractility and weakens wall motion, leading to expansion of infarct and ventricular remodeling. This is extremely prone to HF.^[28,29]^ In the treatment of AMI, timely opening of the affected blood vessels can restore the perfusion in the cardiac muscle, reduce infarct size, and protect left ventricular systolic function. However, some patients still have incomplete blood perfusion in the infarcted area, and this affected area will inevitably cause the deterioration of cardiac function.^[7]^

The LVEF can reflect the contractile capacity and number of functional cardiomyocytes. The lower the LVEF, the lesser the number of functional cardiomyocytes, the greater the proportion of fibrosis and myocardial necrosis, the worse the myocardial contraction, and the worse the patient's prognosis.^[30]^ The lower the LVEF, the higher the mortality rate of HF.^[31]^ Patients with a significantly increased LVEF after treatment have a better prognosis. Our study found that rhBNP increased the LVEF in patients with AMI 1 week, 4 weeks, 12 weeks, and 24 weeks postoperatively, indicating that it can effectively prevent the occurrence of cardiac dysfunction after PCI. Similarly, the recurrence of HF also decreased after PCI in our study.

NT-proBNP is mainly synthesized and secreted by ventricular myocytes. Changes in ventricular volume and wall tension affect its secretion; thus, NT-proBNP accurately reflects changes in early cardiac function.^[32]^ In patients with HF, a decrease in the LVEF and a significant increase in NT-proBNP levels indicate a deterioration in cardiac function and a significant increase in the risk of death. Patients with AMI often have underlying myocardial ischemia, hypoxia, and sympathetic nervous system excitability, which can promote the secretion of Ald due to elevated levels of hormones such as adrenaline, norepinephrine, adrenocorticotropic hormone, and angiotensin. Plasma NT-proBNP and Ald levels in patients with AMI can be used as important biochemical indexes to evaluate the severity of cardiac function after PCI. The results showed that the NT-proBNP levels (24 hours, 48 hours, 72 hours) and Ald levels (24 hours, 72 hours, 168 hours) were significantly decreased in the rhBNP group compared to the control group.

This study preliminarily confirmed that early application of rhBNP on the basis of conventional therapy can effectively improve the cardiac function of patients with AMI undergoing PCI, providing new ideas and evidence for the prevention and treatment of the disease. However, at present, the relevant results are not robust enough to provide a normative guidance for clinical practice due to the risk of bias in the original study. Therefore, it is necessary to carry out multicenter, large-sample, and high-quality studies in the future, so as to clarify the efficacy and safety of rhBNP in the intervention and management of patients with AMI undergoing PCI. Our study updated the previous meta-analysis.^[33]^ The different visiting points have been fully considered for grouped pooling, making the results more clinically valuable and meaningful. On account of the sensitivity analysis and publication bias analysis, we believe that our study was more robust and reliable than the previous one.

### 4.1 Study limitations

It is important to note the limitations to our study and exercise caution while interpreting the results presented here. First, the meta-analysis was limited by the inadequate sample size, particularly in the analysis of HF recurrence. Second, only articles published in English and Chinese language were included; other papers published in other language and other styles such as conferences abstract were missed, which may result in a potential selection bias. Third, the included studies for this meta-analysis were seldom double-blind randomized trials, which may be a major source of selection bias. Fourth, there was significant heterogeneity between included studies, which reduced the reliability of the results. Finally, there were few reports on the safety of rhBNP, and no safety evidence can be drawn. Therefore, future carefully designed studies should be conducted to address these limitations.

## 5 Conclusions

In summary, the application of rhBNP presents a greater clinical benefit to patients with AMI undergoing PCI. Our results might be useful to guide the selection of effective therapy protocols, particularly for patients with AMI undergoing PCI. Nevertheless, due to the limitations previously mentioned, large-scale prospective, randomized trials are warranted to support the results of our current research.

## Acknowledgments

We would like to thank Editage (www.editage.com) for English language editing.

## Author contributions

Cheng Ning, Zhuyuan Fang conceived and designed the experiments. Cheng Ning, Yawei Zheng performed the experiments. Cheng Ning, Yawei Zheng, Jie Li analyzed the data. Cheng Ning, Yawei Zheng, Ming Liu contributed materials/analytical tools. Cheng Ning, Yawei Zheng, Jie Li wrote and revised the manuscript. All authors reviewed and approved the manuscript prior to submission.

ORCID: https://orcid.org/0000-0003-1792-6937
